# Low-dose exposure to PBDE disrupts genomic integrity and innate immunity in mammary tissue

**DOI:** 10.3389/fgene.2022.904607

**Published:** 2022-08-12

**Authors:** Donald M. Lamkin, Shiuan Chen, Karen P. Bradshaw, Shili Xu, Kym F. Faull, Erica K. Sloan, Steve W. Cole

**Affiliations:** ^1^ Norman Cousins Center for PNI, Semel Institute for Neuroscience and Human Behavior, University of California, Los Angeles, Los Angeles, CA, United States; ^2^ Department of Psychiatry and Biobehavioral Sciences, David Geffen School of Medicine at UCLA, Los Angeles, CA, United States; ^3^ Jonsson Comprehensive Cancer Center, University of California, Los Angeles, Los Angeles, CA, United States; ^4^ Department of Cancer Biology, Beckman Research Institute of City of Hope, Duarte, CA, United States; ^5^ Department of Neuroscience, Stanford University School of Medicine, Stanford, CA, United States; ^6^ Department of Molecular and Medical Pharmacology, David Geffen School of Medicine at UCLA, Los Angeles, CA, United States; ^7^ Crump Institute for Molecular Imaging, University of California, Los Angeles, Los Angeles, CA, United States; ^8^ Pasarow Mass Spectrometry Laboratory, Semel Institute for Neuroscience and Human Behavior, University of California, Los Angeles, Los Angeles, CA, United States; ^9^ Monash Institute of Pharmaceutical Sciences, Monash University, Parkville, VIC, Australia; ^10^ Division of Cancer Surgery, Peter MacCallum Cancer Centre-Victorian Comprehensive Cancer Centre, Melbourne, VIC, Austalia; ^11^ Division of Hematology-Oncology, Department of Medicine, David Geffen School of Medicine, University of California, Los Angeles, Los Angeles, CA, United States

**Keywords:** BDE-47, breast cancer, C3(1)/TAg, cGAS-STING, NF-κB, NFE2L2, NHEJ, type I interferon

## Abstract

The low-dose mixture hypothesis of carcinogenesis proposes that exposure to an environmental chemical that is not individually oncogenic may nonetheless be capable of enabling carcinogenesis when it acts in concert with other factors. A class of ubiquitous environmental chemicals that are hypothesized to potentially function in this low-dose capacity are synthesized polybrominated diphenyl ethers (PBDEs). PBDEs can affect correlates of carcinogenesis that include genomic instability and inflammation. However, the effect of low-dose PBDE exposure on such correlates in mammary tissue has not been examined. In the present study, low-dose long-term (16 weeks) administration of PBDE to mice modulated transcriptomic indicators of genomic integrity and innate immunity in normal mammary tissue. PBDE increased transcriptome signatures for the Nuclear Factor Erythroid 2 Like 2 (NFE2L2) response to oxidative stress and decreased signatures for non-homologous end joining DNA repair (NHEJ). PBDE also decreased transcriptome signatures for the cyclic GMP-AMP Synthase - Stimulator of Interferon Genes (cGAS-STING) response, decreased indication of Interferon Stimulated Gene Factor 3 (ISGF3) and Nuclear Factor Kappa B (NF-κB) transcription factor activity, and increased digital cytometry estimates of immature dendritic cells (DCs) in mammary tissue. Replication of the PBDE exposure protocol in mice susceptible to mammary carcinogenesis resulted in greater tumor development. The results support the notion that ongoing exposure to low levels of PBDE can disrupt facets of genomic integrity and innate immunity in mammary tissue. Such effects affirm that synthesized PBDEs are a class of environmental chemicals that reasonably fit the low-dose mixture hypothesis.

## Introduction

The low-dose mixture hypothesis of carcinogenesis proposes that exposure to an environmental chemical that is not individually oncogenic may nonetheless be capable of enabling carcinogenesis when it acts in concert with other factors (e.g., genetic predisposition) (Goodson, 2015). Proponents of this theory assert that cumulative effects of such chemicals over time may persistently disrupt different cancer-relevant pathways, such as those delineated in the Hallmarks of Cancer (Hanahan and Weinberg, 2000), and eventually conspire to induce cancer incidence. The initial presentation of evidence supporting the hypothesis was published in 2015 by an international task force (i.e., the Halifax Project) and included full-length reviews of research linking commonly encountered chemicals to modulation of the biological mechanisms defined in each of the cancer hallmark areas (Harris, 2015).

In their first update to the hallmarks heurisitic, Hanahan and Weinberg (2011) asserted that the capabilities defined in the original core hallmarks are enabled by 1) a succession of alterations in a cell’s genome (i.e., genomic instability) and by 2) the response of innate immunity to an incipient neoplasia (i.e., tumor-promoting inflammation). Evidence continues to accumulate on how genomic instability triggers innate inflammatory responses, with much of the research focusing on the cyclic GMP-AMP Synthase - Stimulator of Interferon Genes (cGAS-STING) pathway (Ablasser and Chen, 2019; Motwani et al., 2019; Decout et al., 2021). DNA damage from exogenous or endogenous factors activates the cGAS-STING pathway, which up-regulates type I interferon (IFN) gene expression and other proinflammatory genes. Although interferon regulatory factor 3 (IRF3) was initially identified as the transcription factor that mediates the type I IFN response to cytosolic DNA (Stetson and Medzhitov, 2006), subsequent research found that canonical nuclear factor kappa B (NF-κB) activity can mediate ∼50% of the response (Abe and Barber, 2014). Thus, the involvement of NF-κB not only amplifies a specific type I IFN response but also transcribes a broader repertoire of proinflammatory genes (Smale, 2010; Balka et al., 2020). Type I IFNs in turn drive the expression of additional IFN stimulated genes (ISGs) by ligating the type I IFN receptor and signaling the transcriptionally active interferon stimulated gene factor 3 (ISGF3) complex (Jefferies, 2019).

Of course, the association between genomic instability and inflammation is not unidirectional. The notion that chronic inflammation can precede a nascent tumor was first suggested long ago by Virchow (Balkwill and Mantovani, 2001), and the modern evidence is now well-organized on how several components of an inflammatory response can contribute to genomic instability and subsequent carcinogenesis (Colotta et al., 2009). Innate immune cells generate reactive oxygen species (ROS) and reactive nitrogen species (RNS) that not only cause DNA lesions at the nucleotide level but also induce single and double strand breaks of the DNA backbone, which potentiate larger-scale mutations, including insertions, deletions, translocations, and sequence rearrangements (Kay et al., 2019).

The Halifax Project identified several environmental chemicals that may interfere with “caretakers of genomic integrity” (i.e., xenobiotic system enzymes and transporters, antioxidant system reductants, DNA damage repair processes) (Langie et al., 2015). Likewise, environmental chemicals were identified that may serve as “immune disruptors,” agonizing tumor-promoting inflammation pathways (Thompson et al., 2015) and/or suppressing anti-tumor immunity (Kravchenko et al., 2015). Listed among the potential immune disruptors that may promote inflammation are synthesized polybrominated diphenyl ethers (PBDEs). Given their flame retardant properties, PBDEs have been incorporated into building materials, plastics, and textiles for use in home construction, electronics, mattresses, automobiles, and numerous other consumer products (Trudel et al., 2011). PBDEs may possibly lower the threshold for antigen to stimulate a proinflammatory response by immune cells (Thompson et al., 2015), as peripheral blood mononuclear leukocyte production of several proinflammatory cytokines (e.g., TNF-α, IL-6) following lipopolysaccaride or phytohaemagglutinin-L stimulation is enhanced by exposure to a commercial mixture of PBDEs (Kronborg et al., 2016). Conversely, other research has found a reduction in stimulated proinflammatory cytokines (i.e., TNF-α, IL-6) by macrophages following exposure to the same PBDE mixture (Hennigar et al., 2012) and a reduction in circulating proinflammatory cytokines/chemokines and innate immune cells *in vivo* following PBDE exposure (Lundgren et al., 2009; Zeng et al., 2014). Despite the down-regulation of inflammatory mechanisms in these latter studies, PBDE was not listed as a potential suppressor of anti-tumor immunity by low-dose theory investigators, although they did note links between inflammation and tumor immunosurveillance (Kravchenko et al., 2015).

In addition to inflammation, PBDEs may affect genomic integrity by impacting cellular defense systems and DNA damage repair processes. Some PBDEs can interact with the key xenobiotic receptor, Aryl hydrocarbon Receptor (AhR), but binding affinities are orders of magnitude below that of the prototypical AhR ligand, dioxin (i.e., TCDD [2,3,7,8-tetrachlorodibenzo-p-dioxin]) (Chen et al., 2001). Moreover, most PBDEs fail to cause transcriptionally active AhR (i.e., the AhR:ARNT heterodimer) to bind the dioxin response element (Chen et al., 2001; Chen and Bunce, 2003). PBDEs have been found to increase oxidative stress (i.e., to increase levels of ROS and lipid peroxidation) (Giordano et al., 2008; He et al., 2008; Costa et al., 2015), which in turn up-regulates the key antioxidant and xenobiotic transcription factor, Nuclear Factor Erythroid two Like 2 (NFE2L2) (Dinkova-Kostova et al., 2002; Levonen et al., 2004; McMahon et al., 2010; Baird and Yamamoto, 2020). Consequently, PBDE-induced oxidative stress has been found to cause DNA damage (Pellacani et al., 2012) and to associate with modulation of DNA damage repair mechanisms in the base excision repair (BER) and homology-dependent recombination (HR) pathways (Gao et al., 2009; Montalbano et al., 2020). Although not tied directly to PBDEs, proteins of the DNA-dependent protein kinase (DNA-PK) complex were recently found to associate with experimental manipulation of STING (Cheradame et al., 2021), strongly suggesting that cGAS-STING signaling from DNA damage may directly regulate the non-homologous end joining (NHEJ) repair pathway (Taffoni et al., 2021).

Given the multiple links between PBDEs and cancer-enabling characteristics of genomic instability and immune disruption, we investigated the effect of low-dose long-term PBDE exposure on transcriptomic indications of genomic integrity and innate immunity in the mammary tissue of mice. Even though chronic PBDE exposure significantly increased indicators of genomic instability, we found that type I IFN and inflammation signaling were significantly down-regulated. We also found that type I IFN signaling was significantly down-regulated in a separate study that examined the effect of PBDE exposure at a higher dose over a shorter term. Finally, we found that replication of the low-dose long-term protocol in transgenic mice that are susceptible to mammary carcinogenesis resulted in greater tumor development.

## Materials and methods

### Long-term PBDE exposure model

To test for effects of prolonged PBDE on genomic integrity and innate immunity in mammary tissue, female nu/nu nude mice were obtained from Charles River Laboratories (Strain #088; Crl:NU-*Foxn1* ^*nu*^). Use of nude mice in the initial long-term experiment enabled additional testing for effects of PBDE on xenograft implantation and growth for a separate study (data not shown). Mice were housed under BSL2 barrier conditions on an individually ventilated cage (IVC) rack in dual filter disposable cages (Innovive, Inc.), with corn cob bedding, nesting tissue for enrichment, and *ad libitum* access to food and water on a 12:12 light:dark cycle at 22°C. The effect of PBDE was examined by administering the BDE-47 congener, the most prevalent PBDE found in humans (Darnerud et al., 2001; Birnbaum and Staskal, 2004), including in the mammary tissue (Petreas et al., 2011). To induce BDE-47 levels in mouse mammary tissue that are similar to elevated levels found in human mammary tissue, we followed a low-dose long-term schedule similar to that described by Koenig et al. (Koenig et al., 2012). After 8 weeks of age, BDE-47 (Millipore Sigma, #91834), in a vehicle of PEG300, 1% DMSO, was administered *via* Alzet osmotic mini-pumps (DURECT Corporation, Model #2006) implanted subcutaneously on the lateral dorsal side of the mouse near the scapula to deliver a dose of 0.03 mg/kg/d for 16 weeks. Control mice received pumps with vehicle alone. Replacement with a second Alzet pump was performed at the half-way point (after 8 weeks) to maintain the dosing schedule (the #2006 model is designed to last for 1.5 times its nominal duration of 6 weeks). All procedures were carried out under protocols approved by the Institutional Animal Use and Care Committee of the University of California, Los Angeles (#2007–155).

### Gas chromatography/mass spectrometry

To measure BDE-47 levels in mouse mammary tissue after 16 weeks of exposure, gas chromatography/mass spectrometry (GC/MS) was utilized. Mammary tissue samples from the 4th mammary fat pad were weighed (range: 10–54 mg) and homogenized at room temperature in a bead mill in methanol with 50 ng of ^13^C_12_-BDE-47 internal standard (IS) (Wellington Labs, #MBDE-47). Homogenates were centrifuged at 25,000 x *g* for 5 min. Duplicate processing for each sample homogenate was then begun as 2 equal volumes of supernatant (800 µL each) were transferred to glass test tubes (10 × 100 mm) and dried in a vacuum centrifuge. Cleanup was accomplished by first dissolving the residuals on the bottom of the test tubes with Milli-Q water (500 µL) and vortexing, followed by ethyl acetate (500 µL) and vortexing, then by sonication in a water bath for 5 min, followed by more ethyl acetate (500 µL) and vortexing. The solutions were centrifuged at 500 x *g* and the ethyl acetate supernatant (500 µL) was transferred to a glass GC injector vial. The lower phase was extracted twice more with ethyl acetate (400 and 200 µL volumes, respectively, using centrifugation without further sonication, as above), and the pooled supernatants were dried in a vacuum centrifuge. Benzene was added (100 µL) and the samples were dried again in a stream of nitrogen. To the thoroughly dried samples N,O-bis(trimethylsilyl)trifluoro-acetamide, containing 10% trimethylchlorosilane (50 μL, v/v), was added, and the samples were incubated (60°C, 60 min) to convert all carboxyl, amino, and hydroxyl functional groups to their corresponding trimethylsilyl derivatives. The derivatized samples were then placed in an autosampler from which an aliquot of each (1 µL) was injected (splitless mode) onto a bonded-phase non-polar fused silica capillary column (Phenomenex ZB-5, phenyl/dimethylpolysiloxane 5/95, 60 m × 0.25 mm, 0.10 µm film thickness; injector port 250°C) and eluted (constant flow, 1 ml/min) with ultra-high purity helium (Thermo Scientific Trace 1310 GC system) over a 63-min temperature ramp (min/°C; 0’/50, 3’/50, 53’/300, 63’/300). The end of the column (GC/MS transfer line at 250°C) was directly inserted into the EI source (180°C, 70 eV) of an Orbitrap mass spectrometer (Thermo Scientific Q Exactive GCMS), calibrated with perfluorotributylamine immediately prior to the analysis of samples scanning from m/z 66–1000 (0.9 s/scan at a resolution [FWHM] of 60,000), with an 8 min solvent delay. Data was collected with instrument manufacturer-supplied software (Thermo Xcalibur). Standards were prepared containing BDE-47 (0, 0.05, 0.5, 5, 50, and 500 ng, all in duplicate) and the internal standard in methanol (750 µL). These samples were processed as described above for the biological samples. Peak areas for BDE-47 at retention time 49.34 min from the reconstructed ion trace at m/z 325.87594 (corresponding to the most intense ion from the (M-Br^+.^) fragment ion) and for ^13^C_12_-BDE-47 at the same retention time from the reconstructed ion trace at m/z 337.91620 (corresponding to the analogous fragment ion) were measured manually with a 5 ppm window using the Qual Browzer subroutine. Data from the standard samples was used to construct a calibration curve, interpolation from which the amount of analyte in each biological sample was calculated.

### Whole transcriptome analysis of mammary tissue

To conduct whole transcriptome analyses of mammary tissue after 16 weeks of exposure to PBDE, mammary tissue samples from the 4th mammary fat pad were homogenized in a bead mill in RLT buffer (Qiagen), and total RNA from RLT buffer lysates was extracted (Qiagen RNeasy Mini Kit, #74104), cleared of contaminating DNA with on-column DNase digestion (Qiagen RNase-Free DNase Set, #79254), and quantified by spectrophotometry (NanoDrop ND-1000; Thermo Scientific). Total RNA (500 ng per sample) was converted to cDNA (Lexogen QuantSeq 3′ FWD) and sequenced using an Illumina HiSeq 4000 instrument in the UCLA Neuroscience Genomics Core Laboratory. Low-level sequencing data was mapped to the RefSeq mouse genome sequence and normalized to counts per million mapped reads (CPM) using the STAR aligner. Transcriptome data from the present study are deposited in the Gene Expression Omnibus (GEO) of the National Center for Biotechnology Information (GSE199054).

To test for group differences in transcriptome signatures of xenobiotic AhR signaling, antioxidant NFE2L2 signaling, DNA damage response signaling, and cGAS-STING signaling, transcriptome representation analyses (TRAs) were conducted (Powell et al., 2013). For each TRA, a transcriptome’s signature was computed for a given subject by calculating the mean expression value, in that subject, of all genes specified as up-regulated in the reference set of the transcriptome in question; down-regulated genes in the reference set were also included but reverse-scored before calculating the mean. Before this mean was calculated for a given subject, all expression values across subjects for a given gene were z-score-transformed to move all genes onto the same scale. The reference set for xenobiotic AhR signaling was composed of genes from MCF-7 breast cancer cells that were differentially expressed by TCDD and evidenced AhR:ARNT binding sites in their promoters (Lo and Matthews, 2012) (Supplementary Data S1). Reference set for antioxidant NFE2L2 signaling was the gene expression set derived by Rooney et al. (Rooney et al., 2020) from multiple NFE2L2 manipulation experiments, including those utilizing the MCF10A breast cell line (Supplementary Data S2). Reference sets for DNA damage repair signaling included the non-overlapping core gene sets defined by Knijnenburg et al. (Knijnenburg et al., 2018) for specific repair pathways that include base excision repair (BER), nucleotide excision repair (NER), mismatch repair (MMR), Fanconi anemia (FA), translesion DNA synthesis (TLS), direct damage reversal/repair (DR), homology dependent recombination (HR), and non-homologous end joining (NHEJ) (Supplementary Data S3). Reference set for cGAS-STING signaling was composed of genes that were up-regulated by cGAS-overexpression independently of canonical IFN signaling (i.e., in cells that were *STAT1*−/−) (Schoggins et al., 2014) (Supplementary Data S4).

To test for a significant difference in activity of the downstream type I IFN and proinflammatory transcription factors ISGF3 and NF-κB, a two-sample variant of the Transcription Element Listening System (TELiS; www.telis.ucla.edu) was used as previously described (Cole et al., 2005). This approach enabled comparisons of the prevalence of binding motifs for these factors in the promoters of genes that were up-regulated and down-regulated by BDE-47 relative to vehicle treatment. Genes that were differentially expressed by BDE-47 were identified based on a > 2-fold biological effect size, rather than statistical effect size (e.g., *t* statistic or *p* value or false discovery rate *q* value), because previous research has shown that biological effect size-based criteria yield more replicable results than do statistical effect size criteria (Cole et al., 2003; MAQC Consortium, 2010) (up-regulated *n* = 1,602; down-regulated *n* = 1,669; Supplementary Data S5). The V$ISRE_01 binding motif definition for ISGF3 and V$NFKAPPAB_01 for NF-κB were retrieved from the TRANSFAC database. Analyses averaged the results derived from nine parametric variations of promoter length (300 bp relative to RefSeq transcription start site, 600 bp, and 1000 bp to +200) and binding motif match stringency (MatSim = 0.80, 0.90, 0.95).

To estimate the percentage of innate immune cell subsets in each sample, the sample’s transcriptome data were deconvolved with the CIBERSORTx support vector regression machine learning approach (https://cibersortx.stanford.edu) (Newman et al., 2015; Newman et al., 2019). This whole-transcriptome digital cytometry was conducted in conjunction with the ImmuCC input matrix that contains murine reference gene expression signatures for innate immune cell types (Chen et al., 2017) (Supplementary Data S6). Innate immune cell populations analyzed were neutrophils, eosinophils, mast cells, resting and activated natural killer (NK) cells, monocytes, resting macrophages (M0), activated M1-like macrophages, activated M2-like macrophages, and immature and activated dendritic cells (DCs).

### Whole transcriptome analysis of short-term PBDE exposure model

To test the generalizability of ISGF3 and NF-κB results in mammary tissue from long-term (16 weeks) PBDE exposure, whole transcriptome data from mammary tissue in a previous smaller study (*N* = 4) of short-term (1 week) PBDE exposure was retrieved from the GEO public repository (GSE125272). Bulk RNAseq data were mapped to the RefSeq GRCm39 mouse genome sequence using the Rsubread package (Liao et al., 2019) and normalized to counts per million mapped reads (CPM). Female BALB/cj mice in the short-term study (Kanaya et al., 2019) were exposed to PBDE at 10 weeks of age by supplementing chow with BDE-47, BDE-100, and BDE-153 to deliver a dose of 1, 0.056, and 0.0126 mg/kg/day. Estrogen was held constant across groups during PBDE exposure, as the mice were ovariectomized at 1 week prior to PBDE exposure and then underwent daily intraperitoneal injection of 17β-estradiol (1 µg/injection) during PBDE exposure. Mammary tissue samples were dissected from the 4th mammary fat pad and included the lymph node. TELiS analysis compared the prevalence of binding motifs for V$ISRE_01 and V$NFKAPPAB_01 in the promoters of genes that were up-regulated and down-regulated by PBDE relative to controls. Differential gene expression was based on a >1.25-fold change in this latter study (up-regulated *n* = 1,579; down-regulated *n* = 1,025; Supplementary Data S7) in order to analyze a number of genes that was comparable to that in the long-term study.

### Mammary carcinogenesis model

To determine whether long-term low-dose PBDE exposure would enhance tumor development in a model of evolving oncogenesis, the C3(1)/TAg transgene model of mammary carcinogenesis was used. Mice express transgene primarily in the distal mammary ductal epithelium and terminal ductal lobular units, leading to changes in genomic organization and the appearance of invasive mammary cancers after 16 weeks of age (Green et al., 2000; Kavanaugh and Green, 2003). Females were obtained from a colony at UCLA that began with hemizygous breeders from The Jackson Laboratory (Strain #013591; FVB-Tg(C3-1-TAg)cJeg/JegJ). When littermates expressing the transgene (genotyping by Transnetyx, Inc.) reached 4–5 weeks of age, they were randomized to receive PBDE or vehicle *via* Alzet osmotic mini-pumps, as described above for nude mice, and allowed to develop tumor. To identify all carcinomas at the conclusion of the exposure period (14–16 weeks), positron emission tomography and computed tomography (PET/CT) were used with the integrated Genisys eight PET/CT scanner (Sofie Biosciences, USA). Mice were anesthetized with 2% isoflurane and injected with 2.6 MBq (70 µCi) of radiotracer, fluorine-18 fluorodeoxyglucose [^18^F]-FDG. After 60 min of probe biodistribution, mice were placed in the imaging chamber and PET images were acquired in listmode for 600 s with an energy window of 150–650 keV and reconstructed using maximum-likelihood expectation maximization (MLEM) as recommended by the vendor. CT imaging was performed immediately after PET imaging. All PET images were corrected for photon attenuation by CT, detector normalization and radioisotope decay, and converted to units of percent injected dose per gram (%ID/g).

### Statistical analysis

**FIGURE 1:**
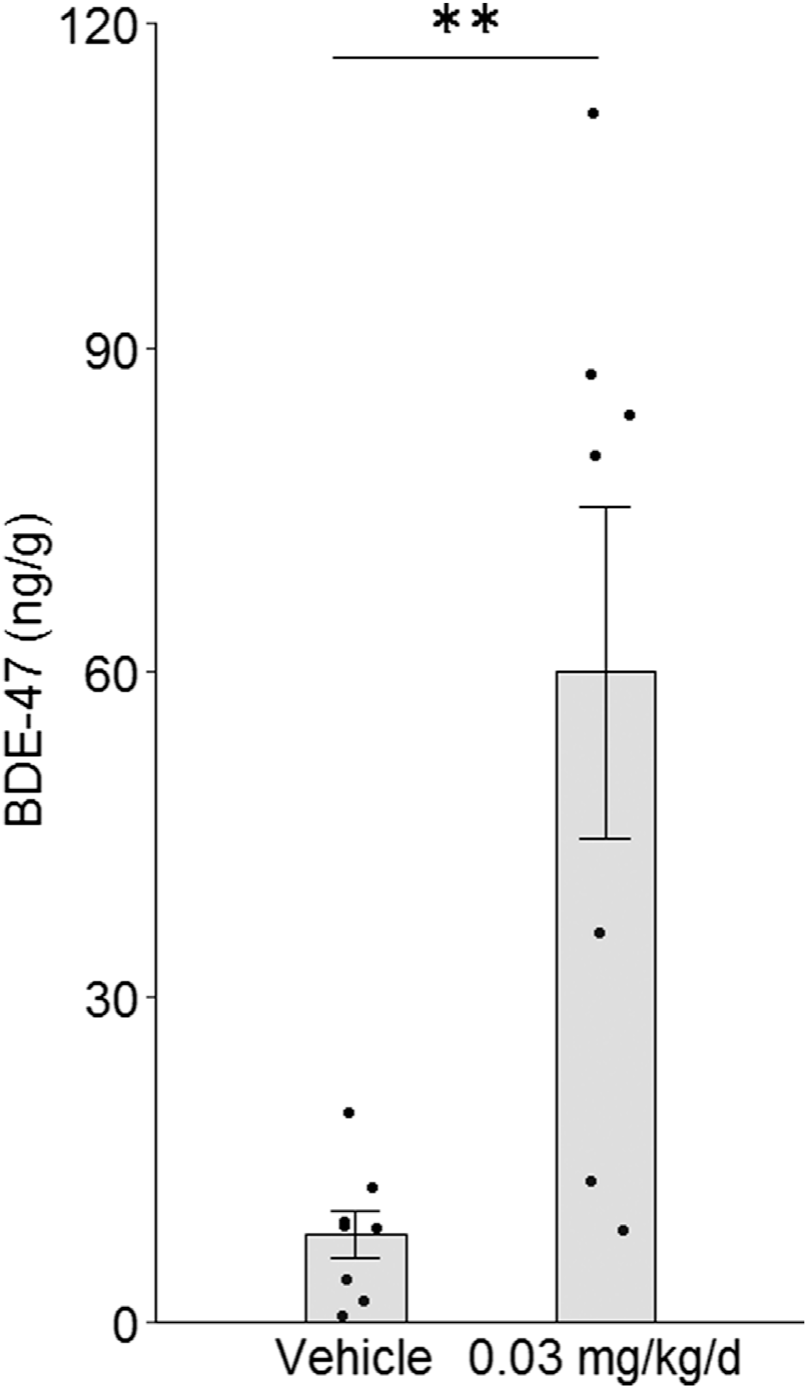
Effect of BDE-47 administration on mammary tissue levels. Mean ± SEM and individual data points for mice receiving 0.03 mg/kg/d of BDE-47 *via* subcutaneous osmotic mini-pump (*n* = 7) or vehicle only (*n* = 8) for 16 weeks **p < 0.01.

**FIGURE 2:**
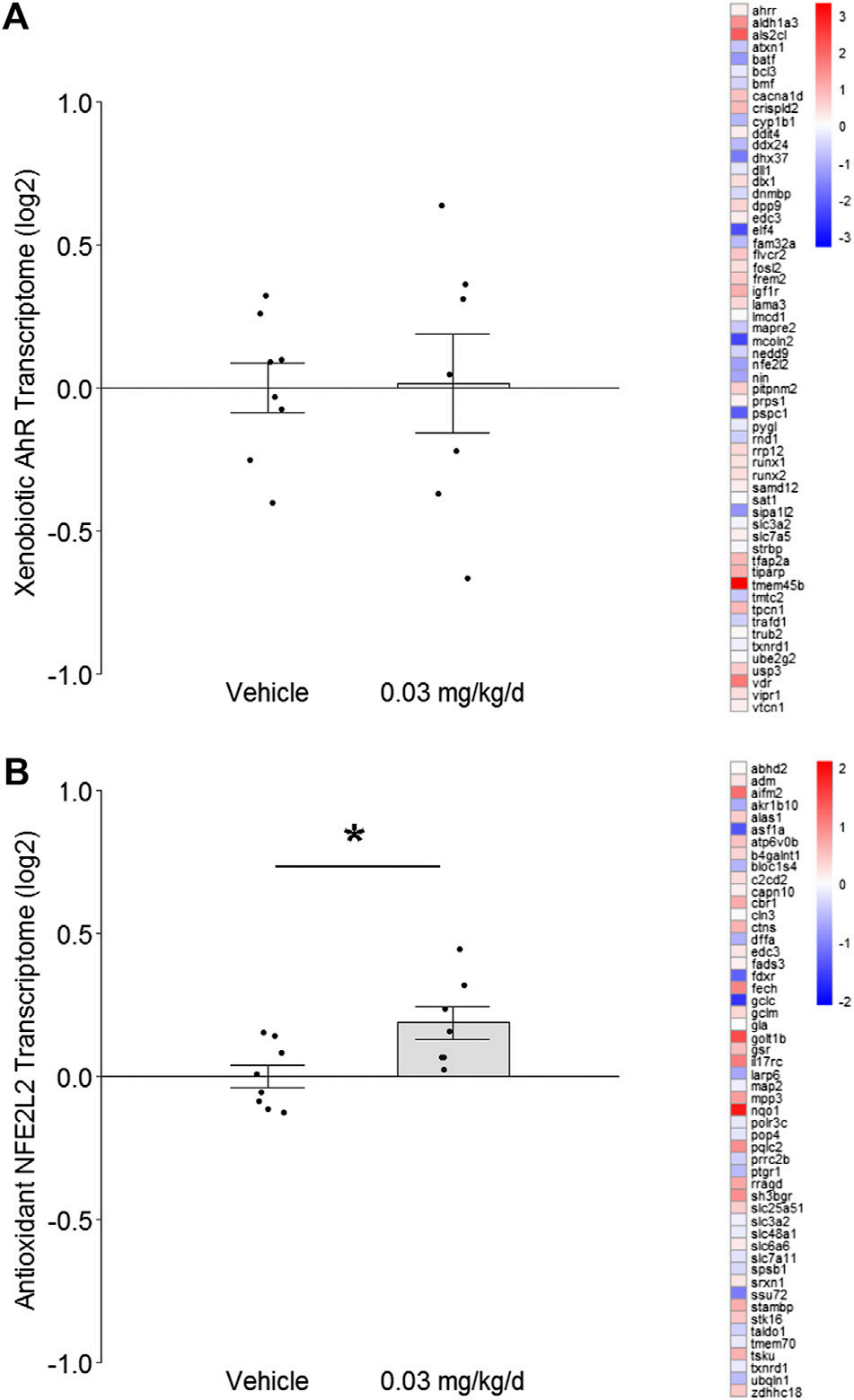
Effect of BDE-47 administration on **(A)** xenobiotic AhR and **(B)** antioxidant NFE2L2 transcriptomes in mammary tissue. Left side: Mean ± SEM with overlay of individual TRA composite score data points, scores on log2 scale centered at zero, *n* = 7-8 mice per group. Right side: Heatmap of mean fold-changes on log2 scale for specific positively expressed genes from the reference transcriptome indicated in the panel. See text for references for gene sets. Complete lists for defining genes for each transcriptome are listed in Supplementary Data S1, S2. *p < 0.05.

**FIGURE 3:**
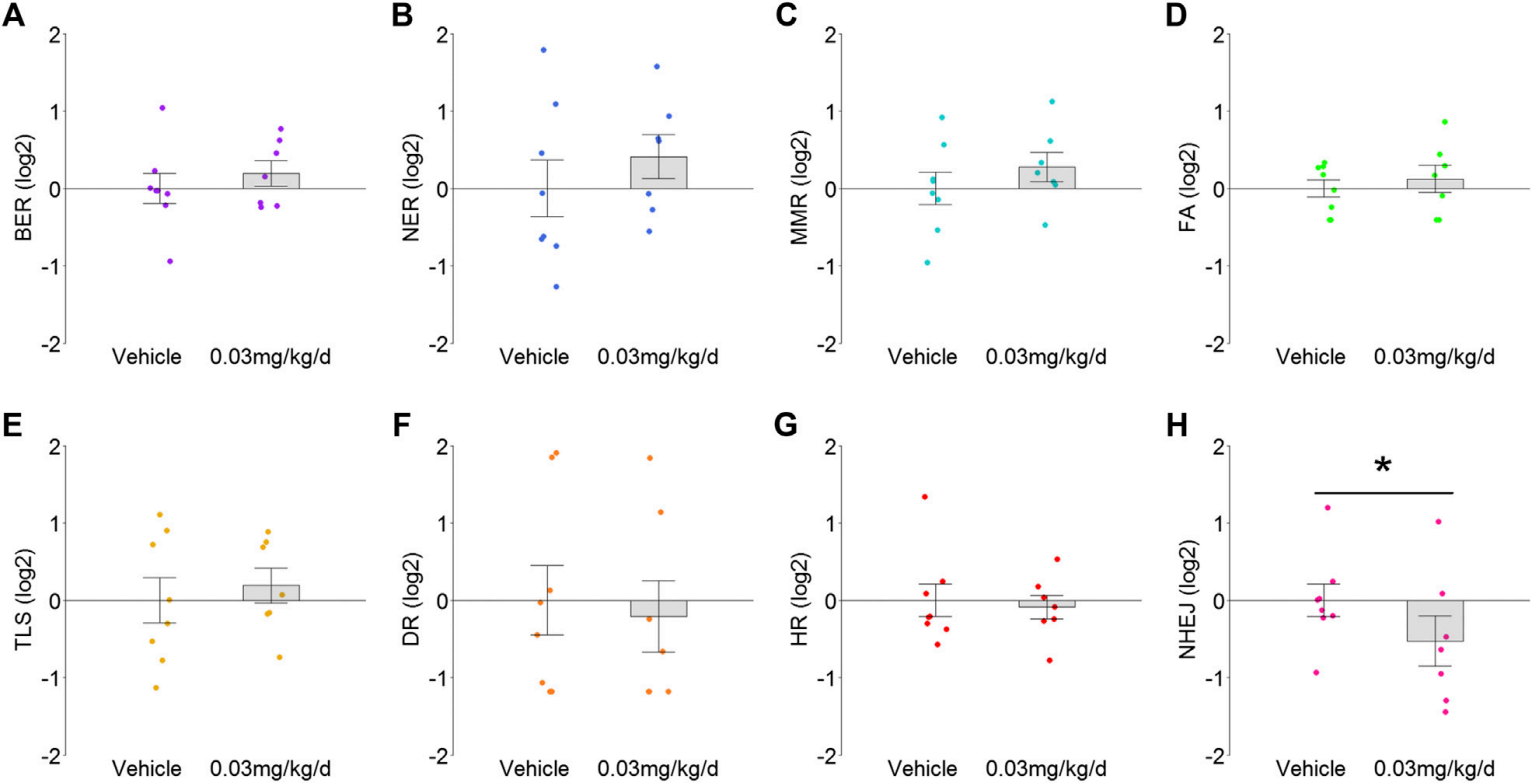
TRA for DNA damage response pathways in mammary tissue after BDE-47 administration for **(A)** base excision repair (BER), **(B)** nucleotide excision repair (NER), **(C)** mismatch repair (MMR), **(D)** Fanconi anemia (FA), **(E)** translesion DNA synthesis (TLS), **(F)** direct damage reversal/repair (DR), **(G)** homology dependent recombination (HR), and **(H)** non-homologous end joining (NHEJ). Mean ± SEM with overlay of individual TRA composite score data points, scores on log2 scale centered at zero, *n* = 7-8 mice per group. See text for reference for gene sets. Complete lists for defining genes for each transcriptome are listed in Supplementary Data S3. *p < 0.05.

**FIGURE 4:**
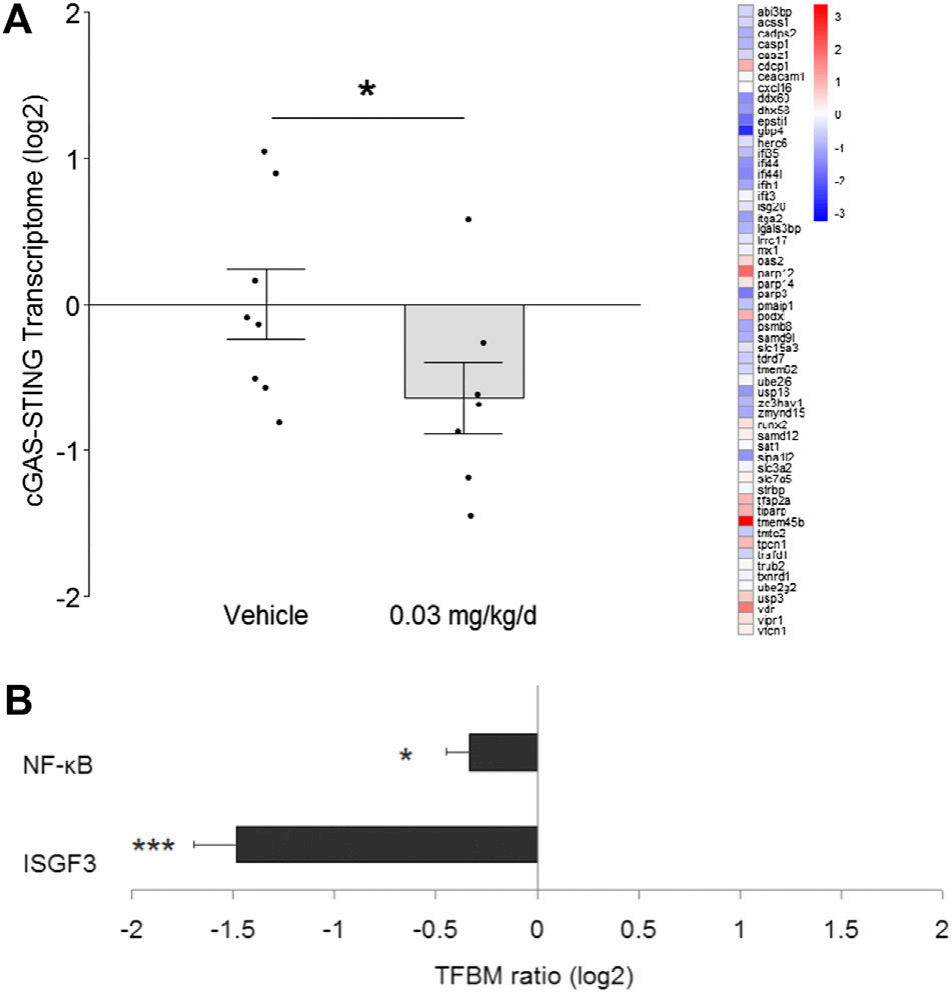
Effect of BDE-47 administration on **(A)** cGAS-STING transcriptome in mammary tissue. Left side: Mean ± SEM with overlay of individual TRA composite score data points, scores on log2 scale centered at zero, *n* = 7-8 mice per group. Right side: Heatmap of mean fold-changes on log2 scale for specific positively expressed genes from the reference transcriptome indicated in the panel. See text for references for gene sets. Defining genes for transcriptome are listed in Supplementary Data S4. **(B)** Effect of BDE-47 administration protocol on proinflammatory signaling pathways in mammary tissue as determined by TELiS analysis of transcription factor bining motifs (TFBM) in promoters of differentially expressed genes listed in Supplementary Data S5. *p < 0.05, ***p < 0.001.

**FIGURE 5:**
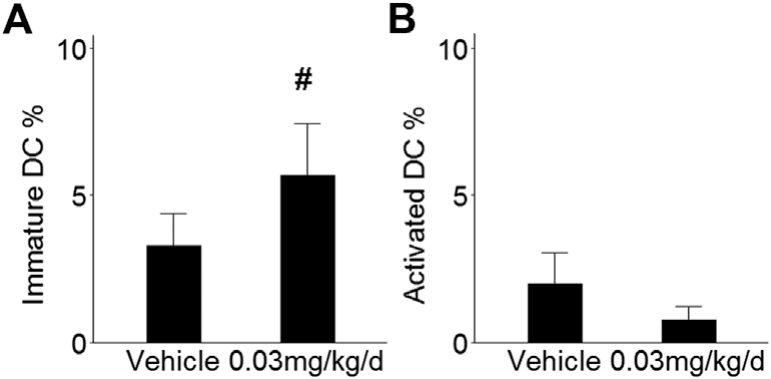
Effect of BDE-47 administration on **(A)** immature DCs and **(B)** activated DCs in mammary tissue as estimated by CIBERSORTx. Mean ± SEM. *n* = 7-8 mice per group. See text for reference for ImmuCC gene set. Defining genes for each cell population are listed in Supplementary Data S6. ^#^ *p* < 0.10.

**FIGURE 6:**
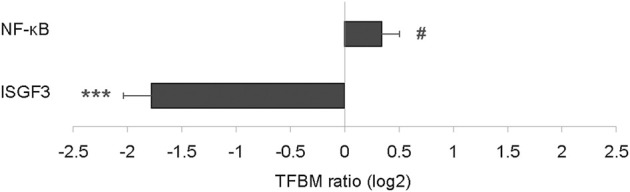
Effect of short-term PBDE administration on proinflammatory signaling pathways in mammary tissue as determined by TELiS analysis of transcription factor binding motifs (TFBM) in promoters of differentially expressed genes listed in Supplementary Data S7. ^#^ *p* < 0.10, ***p < 0.001.

**FIGURE 7:**
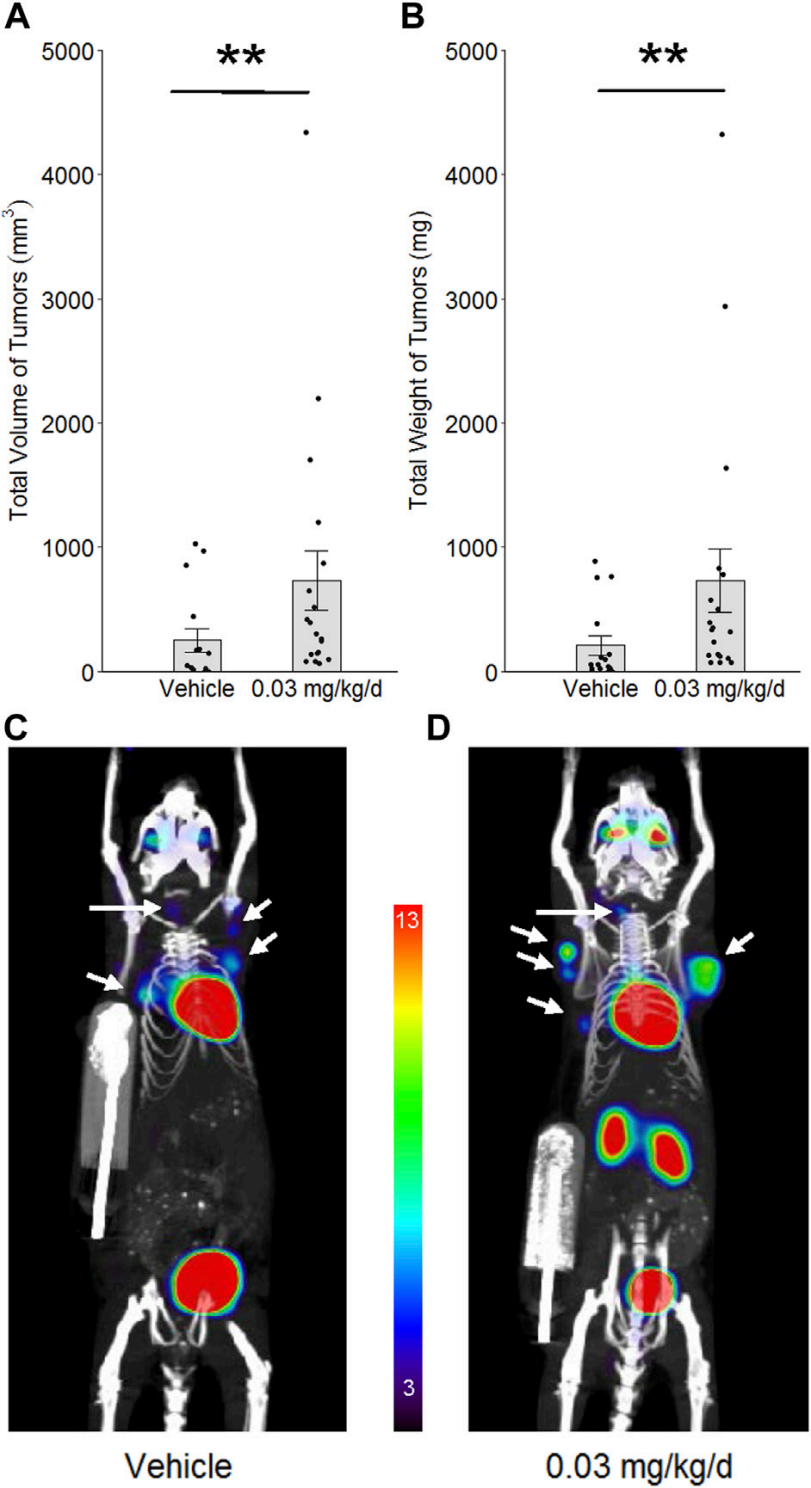
Effect of BDE-47 administration on mammary tumor development in the C3(1)/TAg model. Mean ± SEM and individual data points for **(A)** total volume of tumors and **(B)** total weight of tumors. Representative 10 mm 2D coronal slices, in maximum intensity projection (mip), from 3D PET/CT images of mice randomized to **(C)** only vehicle (*n* = 16) and **(D)** 0.03 mg/kg/d of BDE-47 (*n* = 19). White arrows indicate mammary tumors. Color scale is % ID/g, with threshold set to 3%; note that heart and bladder (and kidneys in the more dorsal 2D slice of panel D) serve as positive control tissue for [^18^F]-FDG in this scan protocol, with % ID/g > 13. **p < 0.01.

Analyses were conducted in the R statistical environment, version 3.6.1 (Core Team, 2019), with additional packages cited below. All distributions were examined for outliers and transformations were applied to normalize distributions where necessary. For Figure 1, Student’s *t*-test was used to analyze the effect of PBDE exposure on mammary tissue levels of BDE-47. For Figures 2–4, analysis of covariance (Singmann et al., 2019) was used to analyze the effect of PBDE exposure on composite scores for each TRA, while controlling for any confounding influence of xenograft presence in the region. For Figure 4B, the mean value of all possible V$ISRE_01 binding motif prevalence ratios and the mean value of all possible V$NFKAPPAB_01 binding motif prevalence ratios were tested for significant deviation from a null population mean ratio of 1 with single sample *t*-test; differentially expressed genes for this analysis were identified while controlling for xenograft. For Figure 5 and Supplementary Figure S1, analysis of covariance was used to analyze the effect of PBDE exposure on CIBERSORTx estimates of innate immune cell populations in mammary tissue, except for those populations that exhibited mostly zero values; for these latter (activated NK cells, activated DCs, M2-like macrophages) logistic regression was used to test significance for the prevalence of non-zero values between groups. Figure 6 used the same approach described for V$ISRE_01 and V$NFKAPPAB_01 for Figure 5. For Figure 7, Student’s *t*-test was used to analyze the effect of PBDE exposure on total volume and total weight of all tumors identified in a given transgenic mouse from the C3(1)/TAg mammary carcinogenesis model.

## Results

### Mammary tissue levels of BDE-47 after long-term PBDE exposure

To induce BDE-47 levels in mouse mammary tissue that are similar to elevated levels found in human mammary tissue, BDE-47 was administered subcutaneously *via* Alzet osmotic mini-pumps to nude mice at a target dose of 0.03 mg/kg/d. Administration of this dose for 16 weeks resulted in a mean mammary tissue level of 60.03 ng/g wet weight (±15.32 SEM) (Figure 1A). This level is on par with previously reported research utilizing a similar low-dose long-term schedule (Koenig et al., 2012) and is representative of the higher-end levels reported in human breast tissue (Petreas et al., 2011). Mice receiving only vehicle also exhibited detectable signal above background, although the signals detected were significantly lower than that of dosed mice by more than 7-fold (*p* = 0.003; Figure 1).

### Genomic integrity and innate immunity in mammary tissue after long-term PBDE exposure

To investigate the effect of prolonged low-dose BDE-47 exposure on genomic integrity safeguards and innate inflammatory responses in mammary tissue, normal tissue was dissected from the left 4th mammary fat pad of nude mice for RNAseq at the end of the 16-weeks BDE-47 administration period. Transcriptome Representation Analyses (TRAs) with reference gene sets for xenobiotic AhR signaling and antioxidant NFE2L2 signaling were conducted to test for indications of cellular stress. Transcriptome signatures for the xenobiotic AhR pathway exhibited no significant difference between groups (*p* = 0.95; Figure 2A). However, transcriptome signatures for the antioxidant NFE2L2 pathway were significantly higher in mice dosed with BDE-47 (*p* = 0.009; Figure 2B).

Given the associations among antioxidant signaling, oxidative stress, and DNA damage, eight distinctly defined DNA damage repair pathways were examined to determine whether they also exhibited modulation. Transcriptome signatures for BER, NER, MMR, FA, and TLS pathways were up-regulated 1.1- to 1.2-fold by BDE-47 administration, but none of these reached statistical significance (*p* values >0.38; Figures 3A–E). In contrast, transcriptome signatures for DR, HR, and NHEJ were down-regulated by BDE-47, with NHEJ exhibiting the largest decrease at 1.4-fold (*p* = 0.03; Figures 3F–H).

To determine whether modulation of DNA damage repair pathways associated with change in cGAS-STING signaling, transcriptome signatures for the latter were analyzed and, similar to NHEJ, found to be significantly down-regulated by 1.6-fold (*p* = 0.048; Figure 4A). Subsequent TELiS analysis of binding motifs for downstream transcription factors ISGF3 and NF-κB indicated that these factors were also significantly down-regulated by 2.8-fold (*p* = 0.0001) and 1.3-fold (*p* = 0.017), respectively (Figure 4B).

To determine whether innate immune cell populations may contribute to the observed effects in mammary tissue, RNAseq data were submitted to the CIBERSORTx platform for digital cytometry. Immature DCs and activated DCs were inversely correlated (r_s_ = -0.78, *p* = 0.0006) and exhibited opposite patterns in mice that received BDE-47: Immature DCs increased by 1.7-fold (*p* = 0.09; Figure 5A), while activated DCs decreased by 2.7-fold (*p* = 0.33; Figure 5B). Remaining innate immune cell populations exhibited unremarkable patterns and were not significantly different between groups (NK cell subsets *p* values >0.82; monocytes/macrophage subsets *p* values >0.71; granulocyte subsets *p* values >0.42: Supplementary Figure S1).

### Confirmatory experiment with short-term PBDE exposure model

To test the generalizability of the ISGF3 and NF-κB results in the long-term model, whole transcriptome data from mammary tissue in an independent experiment (Kanaya et al., 2019) of short-term (1 week) PBDE exposure was analyzed. ISGF3 was, again, significantly down-regulated by 3.4-fold (*p* = 0.0001; Figure 6) after just 1 week of exposure. Conversely, NF-κB was up-regulated by 1.3-fold (*p* = 0.08; Figure 6) at the 1 week time point.

### Carcinogenesis during long-term PBDE exposure

To determine whether PBDE’s effects on genomic integrity and innate immunity associate with enhancement of oncogenesis in mammary tissue, the C3(1)/TAg mouse model of cancer initiation and tumor development was used. Female littermates were randomly assigned to groups in the low-dose long-term protocol at 4–5 weeks of age and allowed to develop tumors until they reached 18–21 weeks of age. By the end of the long-term exposure period, BDE-47 administration increased tumor volume by 3.2-fold (*p* = 0.006) and tumor weight by 3.8-fold (*p* = 0.002; Figure 7).

## Discussion

Prolonged low-dose exposure to PBDE *in vivo* resulted in relative disruption of genomic integrity and innate immunity in murine mammary tissue. This was evidenced by up-regulation of transcriptome signatures for antioxidant signaling, down-regulation of transcriptome signatures for non-homologous end joining DNA repair, and down-regulation of multiple transcriptomic indicators for type I interferon signaling. These systematic genome-wide results suggest that PBDEs—persistent environmental chemicals that readily infiltrate the human body—are capable of significantly altering the intrinsic molecular sentinels that oppose carcinogenesis in mammary tissue.

Previous *in vitro* research has found that PBDEs, including the BDE-47 congener used in the present study, can increase oxidative stress and cause DNA damage (Pellacani et al., 2012). Extending those findings, the present *in vivo* study found that signaling by NFE2L2, which is up-regulated by prooxidants (Dinkova-Kostova et al., 2002; Levonen et al., 2004; McMahon et al., 2010; Baird and Yamamoto, 2020), was significantly increased in mammary tissue by prolonged BDE-47 administration. However, PBDE-induced DNA damage has previously been associated with up-regulation of mechanisms in the homology-dependent recombination (HR) DNA repair pathway (Gao et al., 2009; Montalbano et al., 2020), which functions with the non-homologous end joining (NHEJ) DNA repair pathway to repair double-strand DNA breaks (Mao et al., 2008). In contrast to those previous findings, both the HR and NHEJ pathways were down-regulated by BDE-47 in the present study. The reasons for these disparate findings on DNA damage repair are unclear, although they may include the fact that previous research examined double-strand repair mechanisms on a much shorter time-scale (i.e., ≤72 h) compared to the present study. An alternative explanation is born of the recent finding that STING controls the number of nuclear DNA-PK complex proteins (Cheradame et al., 2021), which characterize the NHEJ repair pathway (Taffoni et al., 2021). Thus, it is plausible that NHEJ repair was down-regulated in the present study because cGAS-STING signaling was down-regulated in the present study.

Findings relating PBDE to immune disruption have been inconsistent, with mitogen-induced proinflammatory cytokine production being either augmented (Kronborg et al., 2016) or attenuated (Hennigar et al., 2012) by PBDE. More consistent are *in vivo* findings that report the near disappearance of several proinflammatory cytokines in peripheral circulation after PBDE exposure, along with a significant reduction of innate immune cells, including neutrophils, monocytes, and dendritic cells (DCs) (Lundgren et al., 2009; Zeng et al., 2014). The present study used digital cytometry to infer the prevalence of such populations in mammary tissue and found higher levels of immature DCs after long-term BDE-47 exposure. Peripheral tissue immature DCs act as steady state sentinels (Mahnke et al., 2002), but stimulation by type I IFN converts them into mature DCs that migrate away toward lymphoid tissue for antigen presentation (Crouse et al., 2015). Thus, a relatively higher number of immature DCs in mammary tissue following prolonged BDE-47 exposure is consistent with the relative absence of type I IFN signaling that was indicated by lower cGAS-STING transcriptome signatures and lower NF-κB and ISGF3 transcription factor activity.

The disconnect found in this study between genomic disruption and decreased type I IFN signaling following long-term PBDE exposure was unexpected. Double-strand DNA damage in live cells from BDE-47 exposure, at least in the short term as noted above (Gao et al., 2009; Montalbano et al., 2020), would be expected to increase cGAS-STING signaling and subsequent inflammation. In line with that expectation, analysis of mammary tissue transcriptome data from relatively short-term PBDE exposure (1 week) found greater proinflammatory NF-κB signaling. However, ISGF3 signaling had already become down-regulated after this relatively short exposure period. The reasons for ISGF3 and, eventually, even NF-κB down-regulation despite ongoing oxidative stress to DNA from prolonged PBDE exposure are unclear. Given that early type I IFN signaling following viral infection can paradoxically induce eventual immunosuppression when the infection persists (Odorizzi and Wherry, 2013; Lee and Ashkar, 2018), it is theorized that a similar negative feedback mechanism may be operating during chronic PBDE exposure. Researchers postulate that negative self-regulation of type I IFN signaling evolved to limit immune-mediated pathology during infections where viral persistence is inevitable (Odorizzi and Wherry, 2013). Thus, if ongoing PBDE-induced DNA damage is triggering similar mechanisms over time to limit collateral damage from inflammation, then the down-regulation in type I IFN signaling found at the conclusion of the exposure period is less surprising.

Replication of the same long-term PBDE exposure protocol in the C3(1)/TAg mouse model of carcinogenesis caused greater tumor development. This latter finding is consistent with the effect of PBDE on type I interferon down-regulation in the prior models. Type I IFNs are required for the immune system’s inhibition of nascent tumor (Dunn et al., 2005), because they enable DC activation and migration during tumor immunosurveillance (Diamond et al., 2011; Fuertes et al., 2011). Thus, less type I IFN signaling from prolonged PBDE exposure would be expected to associate with greater tumor development as found in the latter experiment. However, it is emphasized that this link is only correlational. Future experiments will better delineate the role of type I IFN signaling in PBDE’s effect on carcinogenesis by blocking the type I IFN receptor in C3(1)/TAg mice and by directly administering a type I IFN to such mice. Also, innate immunity notwithstanding, the effects of PBDE on genomic disruption (i.e., more oxidative stress, less NHEJ DNA repair) are not ruled out as potential mediators in this latter experiment with C3(1)TAg mice. NFE2L2 (also known as NRF2) responds to oxidative stress to help prevent cancerous transformation of normal cells but is often up-regulated in established cancers and, thus, can be described as a double-edged sword (Jenkins and Gouge, 2021). As such, it is unclear whether PBDE may have caused NFE2L2 to up-regulate cytoprotective genes in fully transformed cells in C3(1)TAg mice. Less NHEJ repair can associate with greater MMEJ (microhomology-mediated end joining) repair, which in turn is associated with greater genomic instability (McVey and Lee, 2008) and, thus, may have contributed to greater tumor development. Accordingly, more research is needed to further define the links found in the C3(1)/TAg model among chronic PBDE exposure, genomic disruption, relative immunosuppression, and increased mammary carcinogenesis.

Future experiments should aim to improve upon the limitations of the present study. Use of nude mice in the long-term PBDE exposure protocol allowed examination of innate immunity, as it remains intact in these animals (Rossa and D'Silva, 2019), but the use of fully immunocompetent mice in future long-term studies will enable examination of adaptive immunity in PBDE’s effects on normal mammary tissue. Also, the present study suggests that proinflammatory NF-κB signaling is still intact after 1 week of PBDE exposure but becomes significantly down-regulated by 16 weeks of exposure. Future long-term studies that include additional time points will better address the question of when proinflammatory signaling from daily PBDE exposure becomes down-regulated. In the present study, consistent effects on indicators of type I IFN down-regulation, along with enhanced mammary carcinogenesis, suggest that direct tumor immunosurveillance mechanisms may be impaired by PBDE. However, other research suggests that type I IFN signaling may indirectly affect breast cancer through associations with estrogen signaling (Mori et al., 2021). Thus, future experiments that actively probe the role of estrogen in the effect between PBDE and type I IFN signaling during mammary carcinogenesis will be informative for further understanding PBDE’s effect on tumor immunosurveillance. Such experiments will also help disentangle the more direct estrogen-mimicking effects of PBDEs on correlates of mammary carcinogenesis (Kanaya et al., 2019). The present study’s GC/MS assay detected relatively small amounts of BDE-47 in control group mice. Blank samples in the assay gave, in general, very small to non-existent peaks for BDE-47, indicating that the analyte’s presence in control samples does not appear to be a result of laboratory contamination. Although the source of the analyte in control mice is not known, laboratory rodent diets can contain variable levels of persistent environmental contaminants like PBDE (Mesnage et al., 2015). Thus, future experiments will benefit from testing for trace amounts of PBDE in the animal chow and/or other potential environmental sources (e.g., dust in cage bedding) that may build up in mammary tissue over time during a long-term study. Finally, the present study used transcriptomic indications of genomic integrity and innate immunity to examine effects of PBDE in mammary tissue. Future experiments will benefit from additional assays that measure key proteins in these systems to confirm their modulation by PBDE.

In the wake of increasing restriction on PBDE use since the 2000s, manufacturers have turned in large part to other halogenated flame retardants as substitutes, but these latter are thought to pose similar health threats, given their documented bioaccumulation and similar chemical structure to PBDEs (Dodson et al., 2012; Yang et al., 2022). As such, flame retardants that are both halogen-free and biobased have become highly desirable (Chang et al., 2019). Phosphorus- and/or nitrogen-based materials have received much attention in this regard (Thirumal et al., 2010) and have been investigated in various plastics (Lligadas et al., 2006; Chang et al., 2019; Gao et al., 2019) and rigid polyurethane foams (Thirumal et al., 2010; Heinen et al., 2014). Results from those studies indicate that novel biobased flame retardants are a promising alternative to previous options like PBDE, because the biobased agents are not only less toxic to the environment but also similarly effective at flame retardation.

In conclusion, the present research finds that low-dose exposure to PBDE disrupts genomic integrity and innate immunity in mammary tissue. These effects on correlates of carcinogenesis affirm that synthesized PBDEs are a class of environmental chemicals that reasonably fit the low-dose mixture hypothesis.

## Data Availability

The original contributions presented in the study are publicly available. This data can be found here: https://www.ncbi.nlm.nih.gov/geo/, GSE199054.
