# Supplementary material for: Low-dose exposure to PBDE disrupts genomic integrity and innate immunity in mammary tissue
Source: Front Genet. 2022 Aug 12;13:904607. doi: 10.3389/fgene.2022.904607 (PMC9413140; doi:10.3389/fgene.2022.904607)
Supplement: Supplementary file 3 [file Presentation1.PDF]

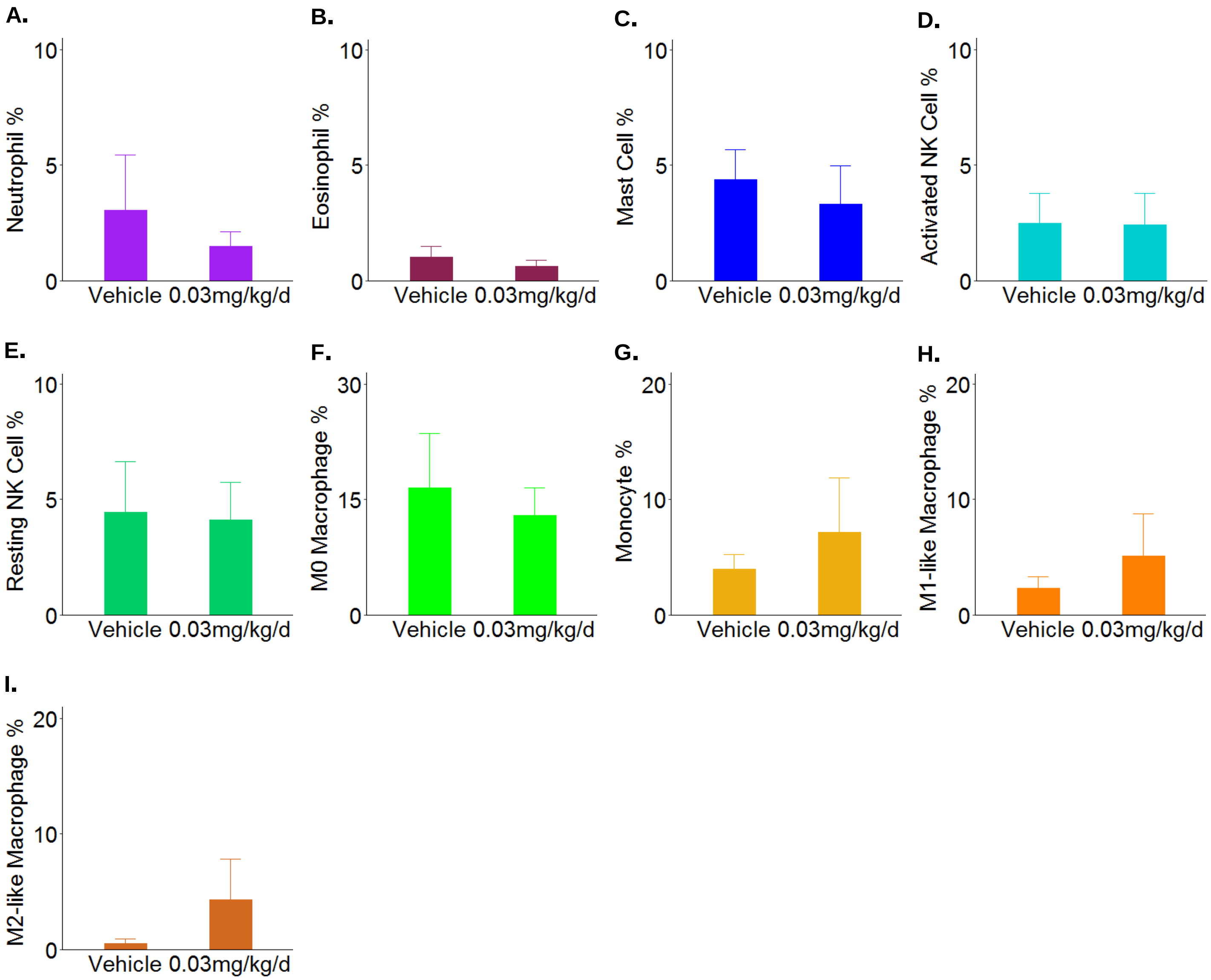

Supplementary Figure 1. Effect of BDE-47 administration on innate immune cell populations in mammary tissue as estimated by CIBERSORTx. Mean ± SEM percentage for (A) neutrophils, (B) eosinophils, (C) mast cells, (D) activated NK cells, (E) resting NK cells, (F) resting M0 macrophages, (G) monocytes, (H) M1-like macrophages, and (I) M2-like macrophages. *n* = 7-8 mice per group. See text for reference for ImmuCC gene set. Defining genes for each cell population are listed in Data S6.
